# Comparative Transcriptomic Analysis of Human Macrophages During *Mycobacterium avium* Versus *Mycobacterium tuberculosis* Infection

**DOI:** 10.1111/mmi.70045

**Published:** 2026-01-05

**Authors:** Gül Kilinç, Robin H. G. A. van den Biggelaar, Tom H. M. Ottenhoff, Leon H. Mei, Anno Saris

**Affiliations:** ^1^ Leiden University Center for Infectious Diseases, Leiden University Medical Center Leiden the Netherlands; ^2^ Department of Biomedical Data Sciences Leiden University Medical Center Leiden the Netherlands

## Abstract

The treatment of *Mycobacterium avium* (*Mav*) infection, responsible for over 80% of nontuberculous mycobacterial pulmonary disease, remains challenging due to rising antibiotic resistance and unsatisfactory success rates. Hence, there is a need for a deeper understanding of host–pathogen interactions to inform the development of alternative therapeutic approaches, like host‐directed therapy (HDT), aimed at improving host antimycobacterial defenses. However, compared to *Mycobacterium tuberculosis* (*Mtb*) infections, knowledge of host–pathogen interactions for *Mav* infection is still limited. To address this knowledge gap, we performed a genome‐wide host transcriptomic analysis of *Mav*‐infected primary human macrophages—the primary host cell—alongside *Mtb*‐infected macrophages to leverage insights from *Mtb* research. Our findings show substantial overlap in the gene expression patterns between *Mav*‐infected and *Mtb*‐infected macrophages, including induction of cytokine responses and modulation of various G‐protein coupled receptors (GPCRs) involved in (lipid‐mediated) macrophage immune functions. Notable differences were observed in the expression of immediate early genes (IEGs), phospholipases, and genes of the GTPase of immunity‐associated protein (GIMAP) family. This study laid a foundation for identifying both shared and *Mav*‐specific host response pathways, providing direction for future investigations into host–pathogen interactions during *Mav* infection and the identification of novel targets for HDT.

## Graphical abstract

Human macrophage transcriptomic responses to *Mycobacterium avium* (*Mav*), a major cause of nontuberculous lung disease, were compared to *Mycobacterium tuberculosis* (*Mtb*). Both infections activated overlapping immune pathways, including cytokine signaling and GPCRs involved in lipid metabolism, while phospholipases were more strongly regulated by *Mav* and IFN signaling by *Mtb*. *Mav* also more strongly regulated GIMAP family genes, cytokines, and immediate early genes, revealing differences in host responses and highlighting potential targets to enhance *Mav*‐specific immunity.

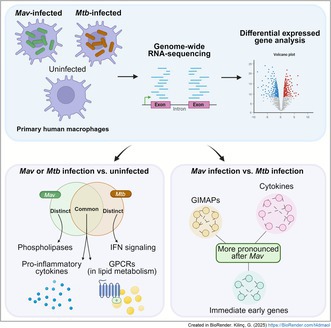

## 1 Introduction

*Mycobacterium avium* (*Mav*) is the causative pathogen for the majority of the chronic lung diseases caused by nontuberculous mycobacteria (NTM) (Ingen et al. 2017; Prevots and Marras 2015; van Ingen et al. 2021), which have seen a rise in incidence globally and represent a growing public health concern (Namkoong et al. 2016; Shah et al. 2016; Winthrop et al. 2020). While lung disease caused by *Mav* (*Mav*‐LD) particularly affects individuals with predisposing lung disorders or a compromised immune system, immunocompetent individuals with certain host characteristics have been found to develop *Mav*‐LD. Improved understanding and management of NTM, in particular *Mav*, infections is therefore desirable.

The recommended treatment for *Mav*‐LD consists of a three‐drug antibiotic regimen comprising a macrolide, ethambutol, and a rifamycin that should be administered for at least 12 months after negative sputum conversion (Daley et al. 2020; Griffith et al. 2007). Nevertheless, even after completing the antibiotic therapy, the success rate, disappointingly, is as low as 40% (Kwon et al. 2019; Xu et al. 2014). This necessitates the development of new therapeutic strategies. One promising approach is the use of host‐directed therapy (HDT), which aims to dampen destructive inflammation or to boost the host's immune responses which may be beneficial, especially for individuals who are suffering from a *Mav* infection and are immunocompromised. By targeting host immunity, HDT may help to eliminate nonreplicating and drug‐resistant bacteria which are hardly eradicated by antibiotic therapy. In addition, as adjunctive treatment, HDT has the potential advantage of shortening the duration or decreasing the dosage of current antibiotic regimens, which may reduce adverse drug effects. Furthermore, since host rather than bacterial pathways are targeted, the risk of de novo development of drug resistance is less likely. While the development of HDT has been extensively explored in the context of tuberculosis (Kilinc et al. 2021; Tian et al. 2025), there remains a notable gap in this area for *Mav* infection. The development of HDT for *Mav* requires a thorough understanding of host–pathogen interactions; however, our current knowledge of these interactions during *Mav* infection remains limited.

Macrophages are the immune cells that play a key role in host defense against *Mav* infection. Upon inhalation, *Mav* enters the lung alveolar space where macrophages will form the main reservoir for the mycobacteria (Crowle et al. 1991; Inderlied et al. 1993). Multiple macrophage receptors, including Toll‐like receptors (TLRs) and C‐type lectins, engage in the initial bacterium‐host cells encounter which induces phagocytosis. Upon recognition and phagocytosis, the early *Mav*‐containing phagosomes ideally undergo maturation and fusion with lysosomes containing hydrolytic enzymes to form phagolysosomes capable of eliminating the mycobacteria (Lee et al. 2020; Uribe‐Querol and Rosales 2017). However, *Mav* produces virulence factors to survive and replicate intracellularly, while evading host immune responses. For instance, the *Mav* protein MAV_2941 inhibits phagosome maturation, and thus prevents intracellular *Mav* killing (Danelishvili et al. 2017; Danelishvili and Bermudez 2015). The production and signaling of pro‐inflammatory cytokines, including TNF, IL‐12, and IL‐23, by macrophages play a vital role in further stimulating the bactericidal functions of macrophages (Park et al. 2022). Consequently, inherited or acquired defects in the production and signaling of these cytokines lead to an increased susceptibility to *Mav*‐LD (Ottenhoff et al. 2002), stressing the significant role of host immunity in deciding the outcome of *Mav* infection.

A better understanding of the mechanisms involved by which macrophages either kill *Mav* or become its breeding ground will aid the development of HDT. RNA‐sequencing (RNA‐seq) has previously been used to study the macrophage host response following infection with *Mtb*, providing insights into the mechanisms of pathogenesis, potential biomarkers for disease progression, and targets for new therapeutic interventions such as HDT (Blischak et al. 2015; Lee et al. 2019; Prombutara et al. 2022; Pu et al. 2021). In contrast, most transcriptomic studies exploring the host response to *Mav* have been conducted in cell lines, which require specific stimulation or may not accurately reflect primary human macrophage responses to mycobacteria and have relied on predefined microarray analyses that fail to reflect the complete transcriptional response (Agdestein et al. 2014; Blumenthal et al. 2005; Greenwell‐Wild et al. 2002; McGarvey et al. 2004). Our aim was therefore to perform genome‐wide transcriptomic analysis of primary human macrophages infected with *Mav*, alongside *Mtb* as a reference to facilitate the rapid extrapolation of relevant findings from *Mtb* to *Mav*, thereby enhancing our understanding of the similarities and differences in how both pathogens interact with and are managed by the host's immune system. We hypothesized that this will ultimately contribute to the development of more effective therapies for infections caused by these mycobacteria.

In this study, we showed that the host transcriptional response is highly similar between macrophages infected with *Mav* and macrophages infected with *Mtb*. The common host response includes the expression of cytokines and other immune‐related genes, but also G protein‐coupled receptors involved in lipid metabolism. Furthermore, we identified genes with transcription levels that were different in magnitude between macrophages infected with *Mav* and macrophages infected with *Mtb*. These differences were linked to phospholipases, immediate early genes (IEGs), and the relatively uncharacterized GTPase of immunity‐associated protein (GIMAP) family.

## 2 Results

### 2.1 Genome‐Wide Transcriptome Analysis of Primary Human Macrophages Infected With *Mav* or *Mtb*

**FIGURE 1:**
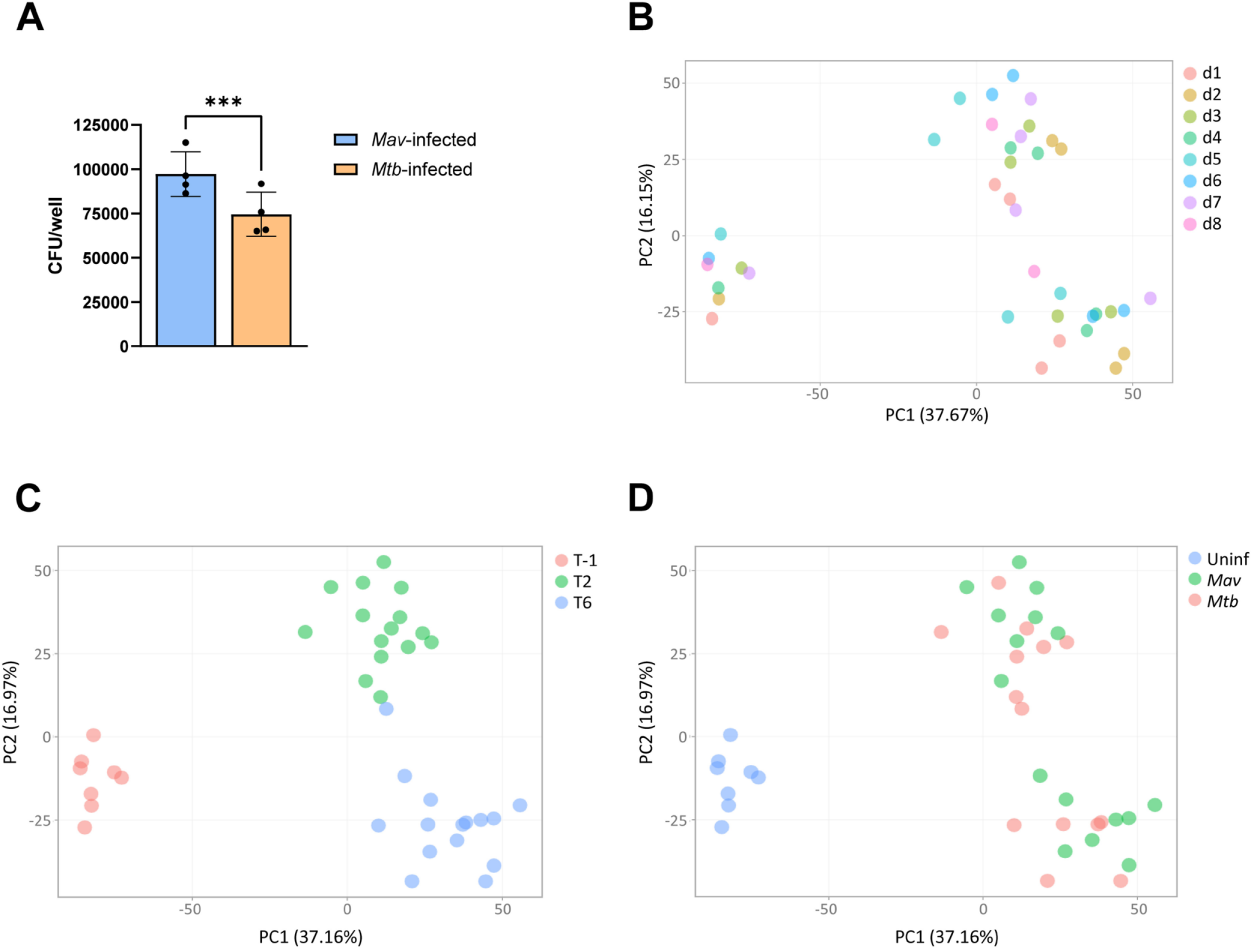
Transcriptome analysis of *Mav*‐ or *Mtb*‐infected versus uninfected samples. (A) M2 macrophages were infected with either *Mav* or *Mtb* for 1 h. After infection, cells were washed and lysed to determine the internalization (T0) by CFU assay. CFU data represent the mean ± standard deviation (SD) from different donors (*n* = 4). Dots represent the mean from triplicate wells of a single donor. Differences were statistically significant by a paired *t*‐test. ***p < 0.001. (B–D) The variance of the sequencing data from *Mav*‐ or *Mtb‐*infected M2 macrophages from different donors (*n* = 8 or *n* = 7, respectively) and uninfected controls was described in PCA plots. Separation of samples in the PCA plots is highlighted by color‐coding the samples according to donor (B), timepoint (C), or infection status (D).

To investigate the induction of the early host immune response, primary human macrophages from 7 donors were infected with *Mav* or *Mtb*, with an 8th donor (*Mtb* data unavailable) maintained in the *Mav* analysis to increase power. Macrophage phagocytosis of *Mav* was modestly higher as compared to *Mtb* (Figure 1A). Moreover, analysis of an independent set of donors confirmed comparable percentages of infected macrophages (Figure S1). Genome‐wide transcriptome analysis using RNA‐seq was performed in seven biological replicates at 2 h and 6 h postinfection. Expression levels were compared between infected samples and uninfected controls using unsupervised and supervised analyses. PCA analysis revealed the clustering of samples derived from different donors (Figure 1B), while infected samples were clustered separately from uninfected macrophages and clearly changed over time (Figure 1C). The transcriptome profiles of macrophages infected with either *Mav* or *Mtb* were evidently clustered together (Figure 1D).

### 2.2 Primary Human Macrophages Infected With *Mav* or *Mtb* Present Similar Host Transcription Responses

**FIGURE 2:**
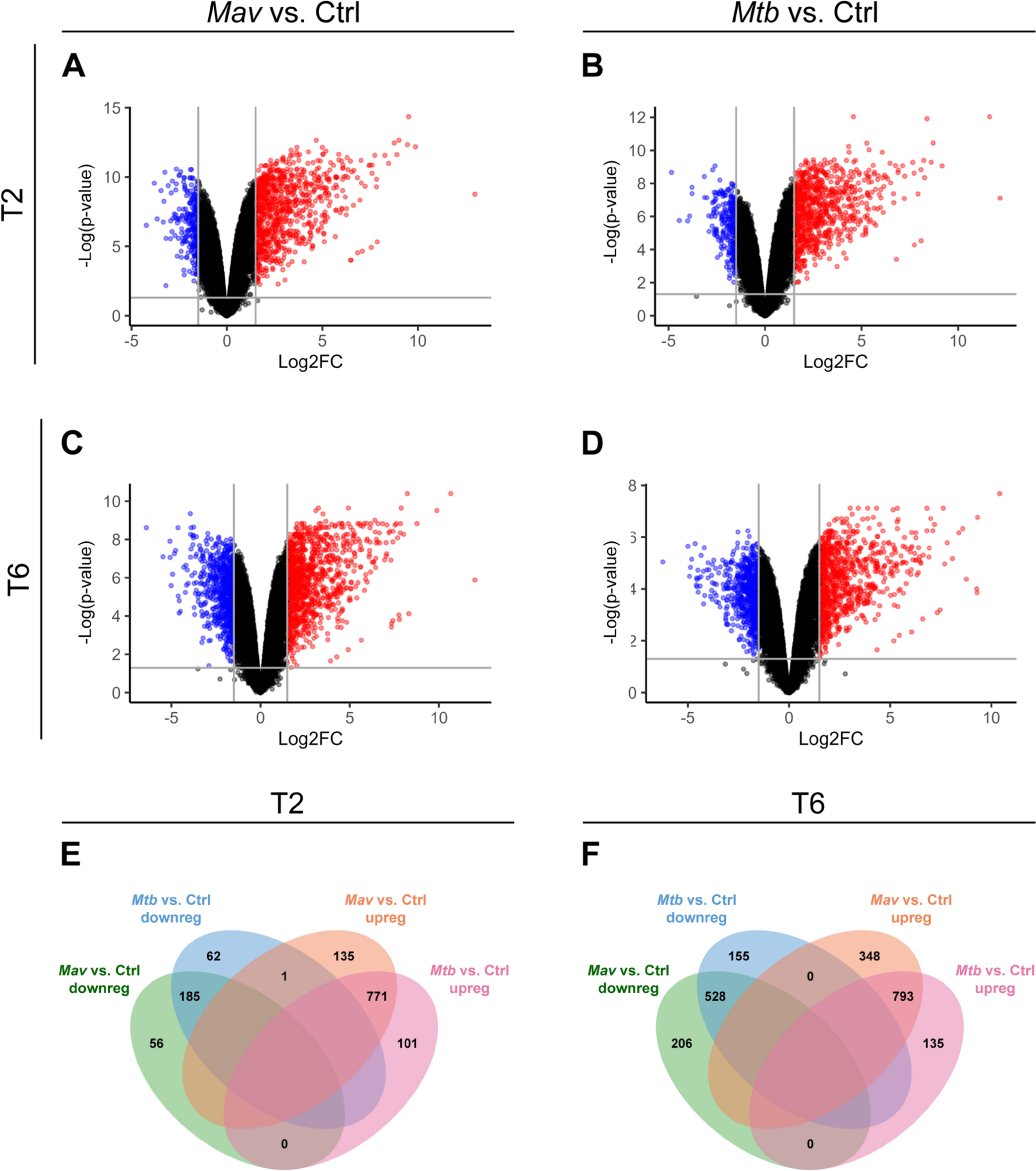
Differential expression analysis of primary human macrophages at 2 and 6 h postinfection with *Mav* or *Mtb* compared to uninfected samples. (A–D) Volcano plots showing DEGs among biological conditions of primary human macrophages at 2 (A, B) or 6 (C, D) hours postinfection with *Mav* (A–C) or *Mtb* (B–D) versus uninfected macrophages (Ctrl). Only log2 fold change (Log2FC) ≥ 1.5 or ≤ 1.5 and false discovery rate‐adjusted *p*‐values < 0.05 were analyzed. The upregulated genes are labeled red and downregulated genes are labeled blue. Nondifferentially expressed genes are labeled black. (E, F) Venn diagram of the DEGs, showing the number of overlapping or unique down‐ or upregulated DEGs identified in macrophages at 2 (E) or 6 (F) hours postinfection infected with *Mav* or *Mtb* compared to the uninfected controls. N/A: Comparison not applicable, as a gene cannot be down‐ and upregulated within the same infection and time point.

To determine the transcriptomic response upon *Mav* and *Mtb* infection, significantly differentially expressed genes (DEGs) (cutoffs: log2(fold change) ≥ 1.5 or ≤ −1.5 and false discovery rate (FDR) adjusted *p*‐values < 0.05) were assessed by comparing gene expression levels in infected macrophages at 2 and 6 h postinfection with uninfected controls. At 2 h postinfection, macrophages showed downregulation and upregulation of 241 and 907 genes after *Mav* infection (Figure 2A, Table S1) or 248 and 872 genes after *Mtb* infection, respectively (Figure 2B, Table S1). At 6 h postinfection, the numbers of downregulated and upregulated genes were 734 and 1141 for *Mav* (Figure 2C, Table S1), and 683 and 928 for *Mtb* (Figure 2D, Table S1), respectively. To compare the similarity between DEGs in response to infection with either *Mav* or *Mtb*, we performed Pearson's correlations and Venn diagram analyses. Changes in gene expression in *Mav*‐ and *Mtb*‐infected macrophages were very similar with Pearson coefficients of determination (*r* ^2^) of 0.95 and 0.93 at 2 and 6 h postinfection, respectively (Figure S2A,B). Correlations between species were stronger than those between two timepoints, with the latter resulting in Pearson correlation coefficients of 0.68 and 0.71 for *Mav* and *Mtb* infection, respectively (Figure S2C,D). Similarly, the Venn diagram analysis showed that the majority of the DEGs was affected by both mycobacteria compared to uninfected controls (Figure 2E,F).

**FIGURE 3:**
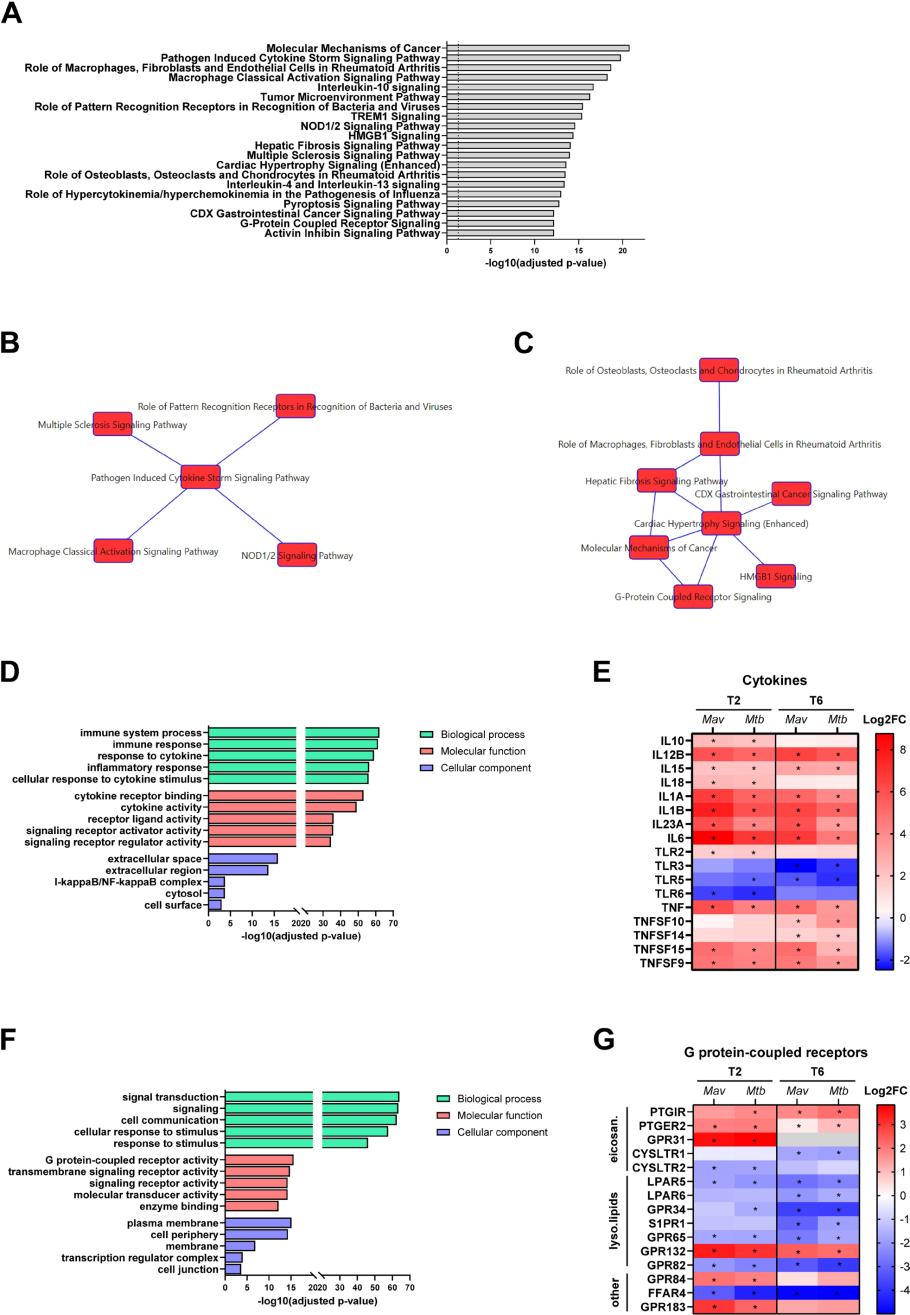
Enrichment analysis of DEGs shared by *Mav* and *Mtb* in primary human macrophages. (A) The top 20 most significantly enriched IPA pathways of the 1673 commonly DEGs in macrophages infected with *Mav* and *Mtb* compared with uninfected controls. The enriched pathways were ranked by −log 10 *p*‐value of gene enrichment. (B, C) Network analysis of enriched pathways from A using the IPA overlap networks tool. Links between indicated pathways indicate an overlap of a minimum of 30 DEGs. (D) GO enrichment analysis showing the top GO terms for biological process, molecular function and cellular component categories enriched for DEGs enriched in the pathways shown in B. The enriched ontology clusters were ranked by log10 *p*‐value of gene enrichment. Asterisk () indicates gene is differentially expressed in comparison to uninfected controls. (E) Heatmap showing the expression patterns of various cytokines that were significantly affected by both *Mav* and *Mtb* infection at 2 (T2) and/or 6 (T6) hours postinfection, in comparison to uninfected controls. Asterisk () indicates a DEG in comparison to uninfected controls. (F) GO enrichment analysis showing the top GO terms for biological process, molecular function and cellular component categories enriched for DEGs enriched in the pathways shown in C. The enriched ontology clusters were ranked by log10 *p*‐value of gene enrichment. (G) Heatmap showing the expression patterns of lipid‐binding GPCRs that were significantly affected by both *Mav* and *Mtb* infection at 2 (T2) and/or 6 (T6) hours postinfection, in comparison to uninfected controls. Asterisk (*) indicates a DEG in comparison to uninfected controls. Grey box indicates no expression values could be determined. Ligands of genes are indicated with eicosan: Eicosanoids, lyso.lipids: Lysophospholipids and other: Free fatty acids and sterols.

To assess the common host response against *Mav* and *Mtb*, DEGs altered by both pathogens at the same timepoint—either 2 h or 6 h postinfection—were pooled, resulting in 610 downregulated genes and 1063 upregulated genes compared to uninfected controls (Table S1). Notably, one gene (*FOS*) was significantly upregulated by *Mav* and downregulated by *Mtb*. The 1673 DEGs shared by *Mav* and *Mtb* were subjected to Ingenuity Pathway Analysis (IPA) (Table S1). The top 20 pathways, enriched with 293 DEGs (17.5% of all DEGs), are shown in Figure 3A. These pathways were also among the highly ranked pathways in response to either *Mav* or *Mtb* compared to uninfected controls (Figure S3A,B). The DEGs enriched in these top 20 pathways showed substantial overlap between pathways, predominantly including cytokines such as *IL1B*, *TNF*, *IL18*, *IL1A*, and *IL6*, as well as *NFKB1* and *NFKB2*. To comprehend the common host response, the overlapping network tool from IPA was used to identify clusters of related pathways. The analyses revealed two major nodes that were affected by both *Mav* and *Mtb* (Figure 3B,C). One node comprised pathways including Multiple Sclerosis Signaling Pathway, Role of Pattern Recognition Receptors in Recognition of Bacteria and Viruses, Pathogen Induced Cytokine Storm Signaling Pathway, Macrophage Classical Activation Signaling Pathway and NOD1/2 Signaling Pathway (Figure 3B). Gene Ontology (GO) Enrichment analysis with the 114 DEGs belonging to this node showed that most of the genes were associated with GO terms linked to a cytokine signaling response (Figure 3D, Figure S4A), which, amongst others, included cytokines (i.e., *CXCL8, CSF2, IL36G, IL12B, IL15, IL10, CCL5, IL23A*), TNF superfamily ligands (*TNFSF10, TNFSF14, TNFSF15* and *TNFSF9*) and Toll‐like receptors (*TLR2, TLR3, TLR5* and *TLR6*) (Figure 3E, Table S1).

The second node comprised pathways including Molecular Mechanisms of Cancer, Role of Macrophages, Fibroblasts and Endothelial Cells in Rheumatoid Arthritis, Role of Osteoblasts, Osteoclasts and Chondrocytes in Rheumatoid Arthritis, Hepatic Fibrosis Signaling Pathway, CDX Gastrointestinal Cancer Signaling Pathway, G‐Protein Coupled Receptor Signaling and HMGB1 Signaling (Figure 3C). GO Enrichment analysis with 164 DEGs (excluding cytokines and cytokine receptors already discussed above) showed an association with mainly signal transduction by G protein‐coupled receptor (GPCR) activity (Figure 3F, Figure S4B). In total, the expression of 39 GPCRs was significantly affected by both *Mav* and *Mtb* (Table S1). Based on the GPCR database (https://gpcrdb.org), a part of these GPCRs are involved in various signaling pathways with ligands including hydrocarboxylic acids (*HCAR2* and *HCAR3*) (Peters et al. 2022; Zandi‐Nejad et al. 2013), neurotransmitters (*CHRM3*), nucleotides (*ADORA2A*, *ADORA3* and *P2RY13*) (Klaver and Thurnher 2021; Jacobson et al. 2018; Aggarwal et al. 2013), hormones (*SSTR2*, *OXTR*, *MAS1*, *MC1R* and *C5AR2*) (Li et al. 2020; Rinne et al. 2017; Hammer et al. 2016; Szeto et al. 2017; Elliott et al. 1999) and Wnt ligands (*FZD2*, *FZD4*, *FZD6* and *LGR4*) (Schaale et al. 2011). Finally, the biggest group comprised GPCRs involved in sensing lipids, including eicosanoids (*PTGIR*, *PTGER2*, *GPR31*, *CYSLTR1* and *CYSLTR2*), lysophospholipids (*LPAR5*, *LPAR6*, *GPR34*, *S1PR1*, *GPR65*, *GPR132* and *GPR82*), free fatty acids (*GPR84* and *FFAR4*) and sterols (*GPR183*) (Figure 3G). Taken together, these findings indicate that common changes in the host transcriptomic response upon infection with *Mav* and *Mtb* are characterized by an enhanced cytokine response, regulation of GPCRs and likely concomitant lipid‐mediated immunoregulation.

### 2.3 Genes Significantly Regulated Only by Either *Mav* or *Mtb* Indicate Subtle, but Not Infection‐Specific, Changes in Host Signaling Pathways

**FIGURE 4:**
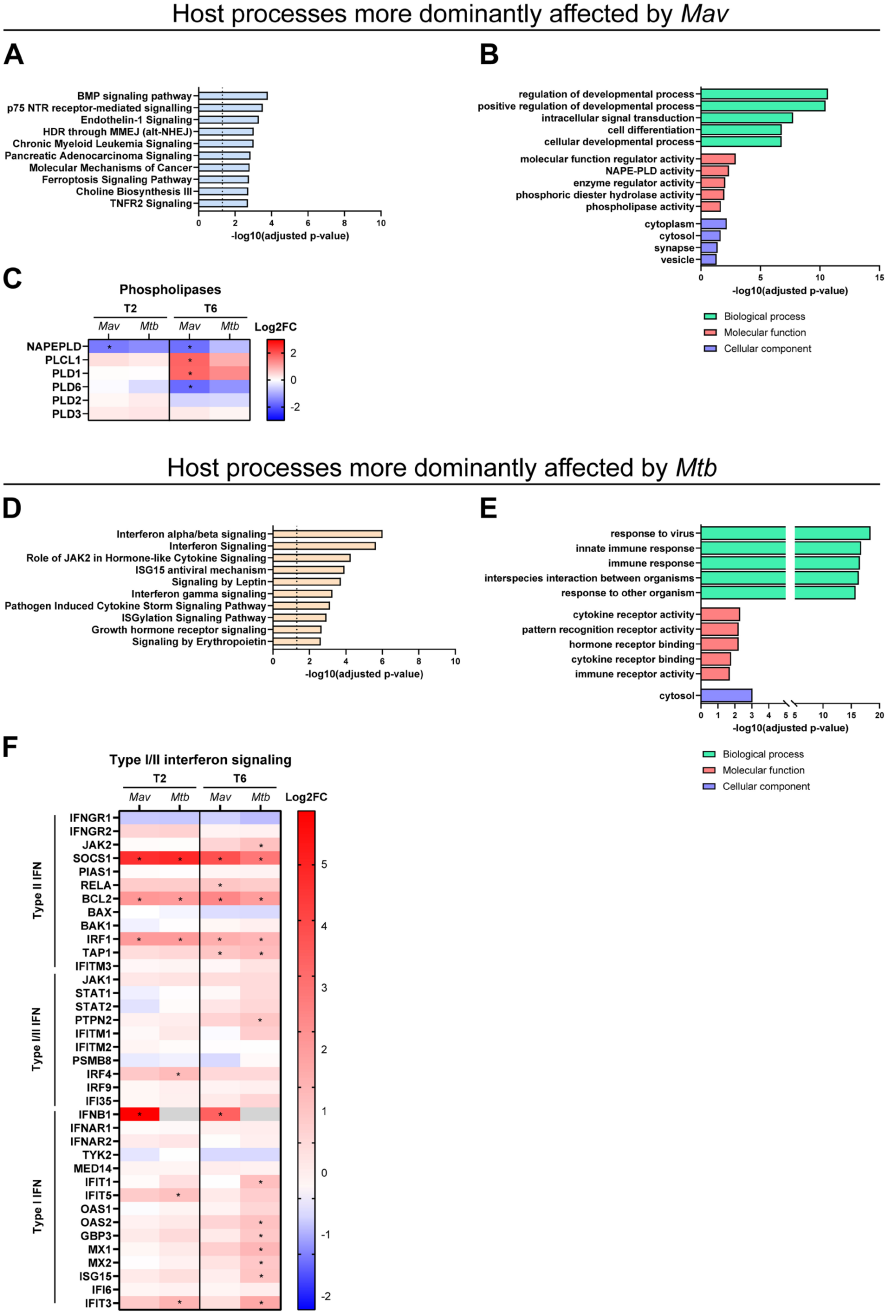
Genes significantly regulated only by either *Mav* or *Mtb* indicate subtle, but not infection‐specific changes in host signaling pathways. (A) The top 10 most significantly enriched IPA pathways of the 561 DEGs in exclusively *Mav*‐infected macrophages compared with uninfected controls. The enriched pathways were ranked by log 10 *p*‐value of gene enrichment. (B) GO enrichment analysis showing the top GO terms for biological process, molecular function, and cellular component categories enriched for DEGs enriched in the pathways shown in A. The enriched ontology clusters were ranked by log10 *p*‐value of gene enrichment. (C) Heatmap showing the expression patterns of phospholipases that were exclusively induced by *Mav* infection at 2 (T2) and/or 6 (T6) hours postinfection, in comparison to uninfected controls, complemented with available expression data of phospholipases which were not affected by infection. Asterisk (*) indicates a DEG in comparison to uninfected controls. (D) The top 10 most significantly enriched IPA pathways of the 323 DEGs induced in exclusively *Mtb*‐infected macrophages compared with uninfected controls. The enriched pathways were ranked by log 10 *p*‐value of gene enrichment. (E) GO enrichment analysis showing the top GO terms for biological process, molecular function, and cellular component categories enriched for DEGs enriched in the pathways shown in E. The enriched ontology clusters were ranked by log10 *p*‐value of gene enrichment. (F) Heatmap showing the expression patterns of type I and II interferon signaling that were exclusively induced by *Mtb* infection at 2 (T2) and/or 6 (T6) hours postinfection, in comparison to uninfected controls, complemented with available expression data of interferon genes which were not detected (grey). Asterisk (*) indicates a DEG in comparison to uninfected controls. Grey box indicates no expression values could be determined.

To identify individual genes that were significantly regulated by either *Mav* or *Mtb*, DEGs from the two different timepoints were pooled. Although the correlation between host transcriptomic response to *Mav* and *Mtb* infection was notably high, genes were identified that were associated with either one of the infections (Figure 2E,F). In total, 561 genes were only differentially expressed by *Mav*, while 323 genes were only differentially regulated by *Mtb* (Table S1). Pathway enrichment analysis revealed that the bone morphogenetic protein (BMP) signaling pathway (*BMP1, BMP2, JUN, MAPK8, RELA, SOS1, RAP1B and PRKAG2*), p75 neurotrophin receptor (NTR)‐mediated signaling (*ARHGEF26, GNA13, ITSN1, MAPK8, PSEN2, RELA, SOS1 and TIAM2*) and TNFR2 Signaling (*BIRC2, JUN, MAPK8 and RELA*) were amongst the most enriched by *Mav* (Figure 4A, Table S1). Importantly, these pathways were not specific for *Mav*, as they were also affected during *Mtb* infections (Figure S5). GO Enrichment analysis with the 39 DEGs enriched in the top 10 pathways affected after *Mav* identified a potential more dominant role of phospholipases during *Mav* infection (Figure 4B, Table S1). We observed that the expression of NAPE‐PLD and *PLD6* (phospholipase D6) was significantly downregulated, while *PLCL1* (phospholipase C like 1) and *PLD1* (phospholipase D1) were significantly upregulated by *Mav* and not by *Mtb* (Figure 4C). Interestingly, in response to both *Mav* and *Mtb*, we observed a significant downregulation of *FFAR4* (Table S1), described to reduce lipid accumulation in macrophages (Zhang et al. 2024). These observations suggest that host lipid metabolism is important for both mycobacteria, as is well known for *Mtb* (van der Klugt et al. 2024).

The genes that were significantly affected by *Mtb* were enriched in pathways associated with an immune response characterized by interferon‐alpha/beta (*IFIT5, IFIT1, IFIT3, IRF4, ISG15, MX1*, and *MX2*) and interferon‐gamma (*GBP3, IRF4, JAK2, OAS2, PTPN2*, and *TRIM5*) signaling pathways, as well as interferon‐stimulated gene 15 (ISG15) signaling (*IFIT1, MX1, MX2, DTX3L, HERC5, IRF4, ISG15, ITGA2*, and *RIGI*) (Figure 4D). GO Enrichment analysis with the 29 DEGs enriched in the top 10 pathways after *Mtb* infection showed that these genes were associated with signaling in response to pathogens, consisting of mainly type I and type II interferon responses (Figure 4E). Like *Mtb*, *Mav* stimulated the expression of genes involved in interferon signaling (Figure 4F). This observation is reflected by the fact that these pathways were enriched among the transcriptomic response to both *Mav* and *Mtb* infections (Figure S5). However, while *Mtb* evoked both type I and type II interferon signaling, *Mav* mainly affected type II interferon signaling. An exception was *IFNB1*, which was solely induced upon *Mav* infection.

### 2.4 Genes Differentially Expressed in Macrophages Infected With *Mav* Compared to *Mtb* Are Associated With Lipid Metabolism, Immediate Early Genes, and the GIMAP Family

**FIGURE 5:**
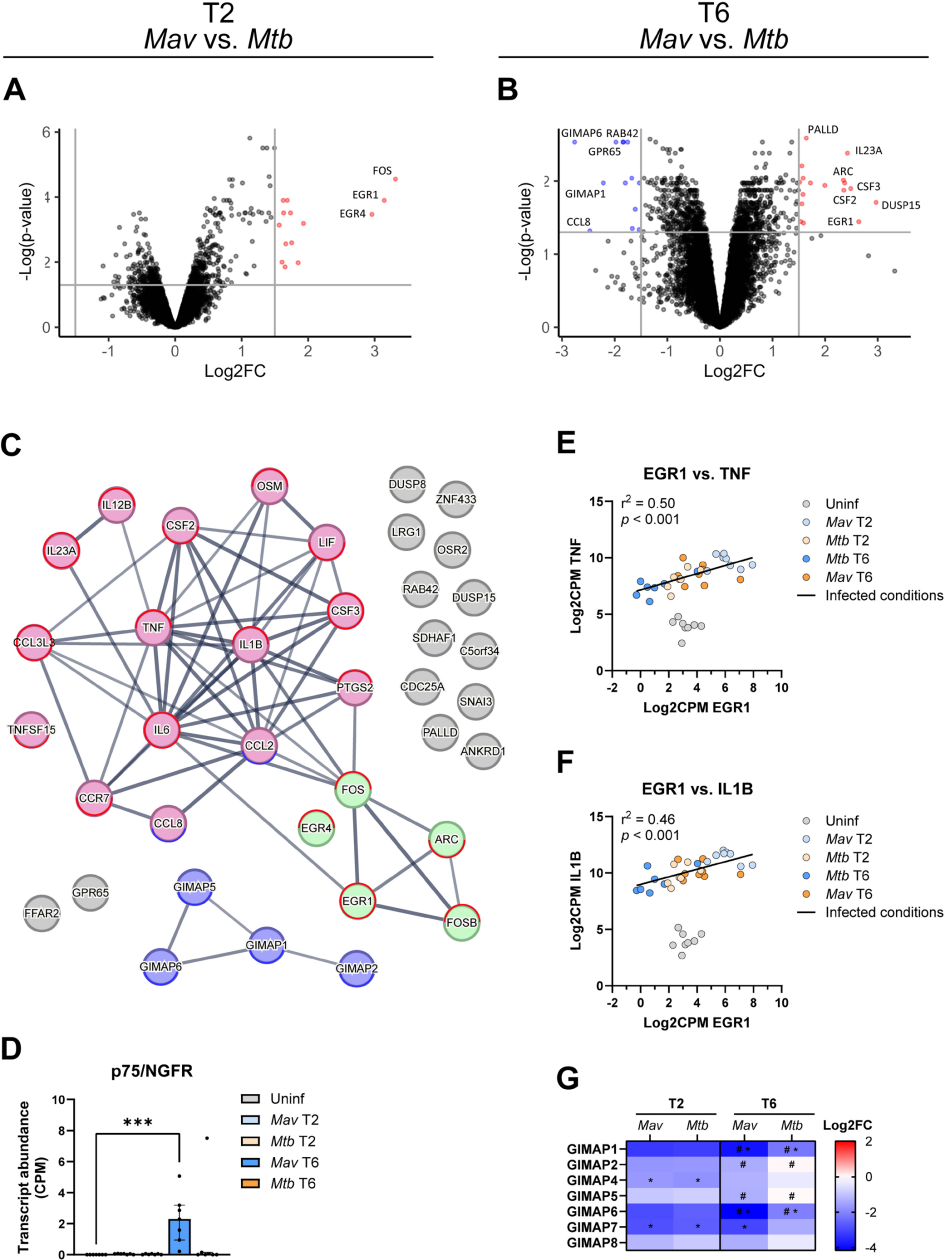
Genes differentially expressed in macrophages infected with *Mav* compared to *Mtb*. (A, B) Volcano plots showing DEGs among biological conditions of primary human macrophages at 2 (A) or 6 (B) hours postinfection with *Mav* versus *Mtb* (*n* = 7). Only log2 fold change (Log2FC) ≥ 1.5 or ≤ 1.5 and false discovery rate‐adjusted *p*‐values < 0.05 were analyzed. The upregulated genes are labeled red and downregulated genes are labeled blue. Nondifferentially expressed genes are labeled black. (C) PPI network showing the DEGs from *Mav*‐infected macrophages compared with *Mtb*‐infected macrophages from A, B. The color representation indicates three distinct networks. Outline of genes indicates expression is increased (red) or decreased (blue), at 2 h (upper circle) or 6 h (lower circle), or both timepoints (full circle), postinfection with *Mav* compared to *Mtb* infection. (D) Transcript levels (count per million; CPM) of *NGFR* in uninfected (grey), and *Mav* (blue shaded)‐ and *Mtb* (orange shaded)‐infected macrophages at 2 and 6 h postinfection. Differences were statistically significant by a Friedman test with Dunn's multiple comparison test. ***p < 0.001. (E‐F) Correlations between log2‐transformed transcript levels (counts per million; CPM) of *EGR1* vs. *TNF* (E) or *IL1B* (F). The *r* ^2^ describes the Pearson coefficients of determination between genes for infected conditions (all samples except those uninfected). (G) Heatmap showing the expression patterns of GIMAPs that were differentially regulated in *Mav*‐infected macrophages at 2 (T2) and/or 6 (T6) hours postinfection, compared to uninfected or *Mtb*‐infected macrophages, complemented with available expression data of GIMAPs which were not affected by infection. Asterisk (*) indicates differential expression when compared to uninfected controls, whereas number sign (#) indicates differential expression between *Mav* and *Mtb*.

In the previous analyses, we focused on the DEGs that were identified relative to uninfected controls. In the following analysis, the magnitude of gene expression was compared between the two infections to uncover significant changes between *Mav* and *Mtb* that may have been overlooked in comparison with uninfected controls. At 2 h postinfection, this comparison revealed 14 genes that were significantly upregulated by *Mav* compared to *Mtb* and no genes that were downregulated in *Mav* (Figure 5A, Table 1; Tables S1 and S2). At 6 h postinfection, *Mav* infection resulted in 13 DEGs with downregulated expression levels and 17 DEGs with significantly upregulated expression levels compared to *Mtb* infection (Figure 5B, Table 1; Tables S1 and S2). Protein–protein interaction (PPI) network analysis using the Search Tool for the Retrieval of Interacting Genes (STRING) database identified three distinct interaction networks including 24 of 38 genes: IEG transcription regulators, GIMAPs, and cytokines (Figure 5C). Interestingly, among the genes that were not associated with a network, *FFAR2* and *GPR65* are related to lipid binding and/or metabolism and were significantly higher expressed in *Mav*‐infected macrophages compared to those infected with *Mtb* (Table S2) (Al Mahri et al. 2022; Lin et al. 2017; Xu et al. 2024).

**TABLE 1:** Genes, belonging to one of the STRING nodes, differentially modulated in primary human macrophages in response to *Mav* compared to *Mtb*.

2 h postinfection	Log2FC (*Mav* vs. *Mtb*)	DEG vs. uninfected		
Gene		*p*‐value (adj)	*Mav*	*Mtb*
**Cytokines/Chemokines**				
IL23A	1.85	1.05E‐02	Up	Up
IL6	1.75	2.52E‐03	Up	Up
OSM	1.74	3.06E‐04	Up	—
IL1B	1.69	1.26E‐04	Up	Up
IL12B	1.65	1.40E‐02	Up	Up
CCL3L3	1.63	3.06E‐04	Up	Up
TNF	1.62	1.26E‐04	Up	Up
CSF3	1.57	7.26E‐04	Up	Up
**Transcription regulators**				
FOS	3.31	2.80E‐05	Up	Down
EGR1	3.14	1.26E‐04	Up	—
EGR4	2.96	3.39E‐04	Up	—
FOSB	1.93	6.47E‐04	Up	—
**Other**				
PTGS2	1.66	2.72E‐03	Up	Up

6 h postinfection	Log2FC (*Mav* vs. *Mtb*)	DEG vs. uninfected		
Gene		*p*‐value (adj)	Mav	Mtb
**GIMAPs**				
GIMAP1	−2.21	1.06E‐02	Down	Down
GIMAP2	−1.66	4.45E‐02	—	—
GIMAP5	−1.80	1.06E‐02	—	—
GIMAP6	−2.76	2.93E‐03	Down	Down
**Cytokines/Chemokines**				
CCL8	−2.47	4.79E‐02	—	Up
CCL2	−1.67	9.12E‐03	—	—
CSF3	2.49	1.26E‐02	Up	Up
IL23A	2.42	4.15E‐03	Up	Up
TNFSF15	2.35	1.33E‐02	Up	Up
CSF2	2.35	9.73E‐03	Up	Up
IL6	1.99	1.15E‐02	Up	Up
CCL3L3	1.58	1.52E‐02	Up	Up
CCR7	1.56	2.05E‐02	Up	Up
LIF	1.52	9.98E‐03	Up	Up
**Transcription regulators**				
EGR1	2.64	3.61E‐02	—	Down
**Other**				
ARC	2.37	1.06E‐02	Up	—

The first network consisted of IEGs *FOS*, *FOSB* (AP‐1 transcription factor complex), *EGR1*, *EGR4* (EGR family of transcription factors), and *ARC*, which were all found to increase after *Mav* infection relative to *Mtb* infection (Table 1). IEGs play key roles in regulating various biological processes including cell proliferation, differentiation and survival, and the production of pro‐inflammatory cytokines IL‐1β and TNF (Bahrami and Drablos 2016; Silverman et al. 2001; Yan et al. 2000). *EGR1*, *EGR4*, *FOS, FOSB* and *ARC* are part of the Reactome pathway of nerve growth factor (NGF)‐stimulated transcription (R‐HSA‐9031628). NGF signaling involves a high‐affinity receptor, TrkA, and a low‐affinity receptor, p75/*NGFR*, which upon activation either induce cell survival or apoptosis, respectively (Bruno et al. 2022; Dechant and Barde 2002). Expression of the p75/*NGFR* gene was low at baseline but became significantly upregulated 6 h postinfection with *Mav*, in line with upregulation of its signaling pathway (Figures 4A and 5D). Therefore, we investigated a potential link with pro‐apoptotic gene regulation by examining the expression of additional key apoptosis markers. The anti‐apoptotic gene *BCL2* was significantly upregulated, particularly in Mav‐infected macrophages while the pro‐apoptotic gene *BAX* was significantly downregulated in macrophages infected with either *Mav* or *Mtb* (Figure S6A,B), actually suggesting a shift towards host cell survival rather than apoptosis, especially in *Mav*‐infected cells. Given the role of IEGs in the production of pro‐inflammatory cytokines, we next investigated whether their expression correlated with transcription of pro‐inflammatory cytokines. Indeed, *EGR1* expression correlated well with *TNF* (*r* ^2^ = 0.50, *p* < 0.001; Figure 5E), *IL1B* (*r* ^2^ = 0.46, *p* < 0.001; Figure 5F) as well as *IL6* (*r* ^2^ = 0.44, *p* < 0.001). Together, these findings suggest that IEG upregulation in *Mav*‐infected macrophages is associated with increased pro‐inflammatory cytokine responses.

The second network consisted of genes of the GIMAP family, which were significantly downregulated in macrophages infected with *Mav* compared to those infected with *Mtb*. *GIMAP1* and *GIMAP6* showed reduced expression in macrophages 6 h postinfection with *Mav* and *Mtb* compared to uninfected controls, with significantly more silencing by *Mav* compared to *Mtb* (Figure 5G). Although *GIMAP5* and *GIMAP2* were not significantly affected by mycobacterial infection when compared to uninfected controls, these genes were downregulated in macrophages infected with *Mav* compared to *Mtb*. Furthermore, while not differentially regulated between the two mycobacteria, *GIMAP4* and *GIMAP7* were significantly silenced by both *Mav* and *Mtb* 2 h postinfection in comparison to uninfected controls.

Finally, the third PPI network consisted of genes encoding mainly cytokines. While we observed that both *Mav* and *Mtb* triggered significant early cytokine responses in macrophages compared to noninfected controls, *Mav* induced a more pronounced upregulation of several cytokines compared to *Mtb*. At 2 h postinfection, these cytokines included *IL23A*, *IL6*, *IL1B*, *IL12B*, *CCL3L3*, *TNF* and *CSF3* (Table 1). At 6 h postinfection, the upregulation of *IL23A*, *IL6*, *CCL3L3*, and *CSF3* persisted, along with the downregulation of *CCL8* and *CCL2* and additional upregulation of cytokines *TNFSF15*, *CSF2*, and *CCR7* in response to *Mav* compared to *Mtb* (Table 1, Figure 5C). The heightened expression of these molecules in response to *Mav* suggests this infection might be stimulating a more intense or swifter activation of immune pathways compared to *Mtb*. In addition, macrophages infected with *Mav* or *Mtb* showed increased expression of *PTGS2*, which was significantly higher upon *Mav* compared to *Mtb* infection.

Taken together, macrophages infected with *Mav* showed upregulation of pro‐inflammatory cytokines compared to *Mtb* infection, which was correlated with increased expression of IEG transcription factors, whereas transcription of GIMAP genes was downregulated.

### 2.5 Validation of Upregulated Cytokine Expression by Assessing Cytokine Secretion by *Mav*‐ and *Mtb*‐Infected Macrophages

**FIGURE 6:**
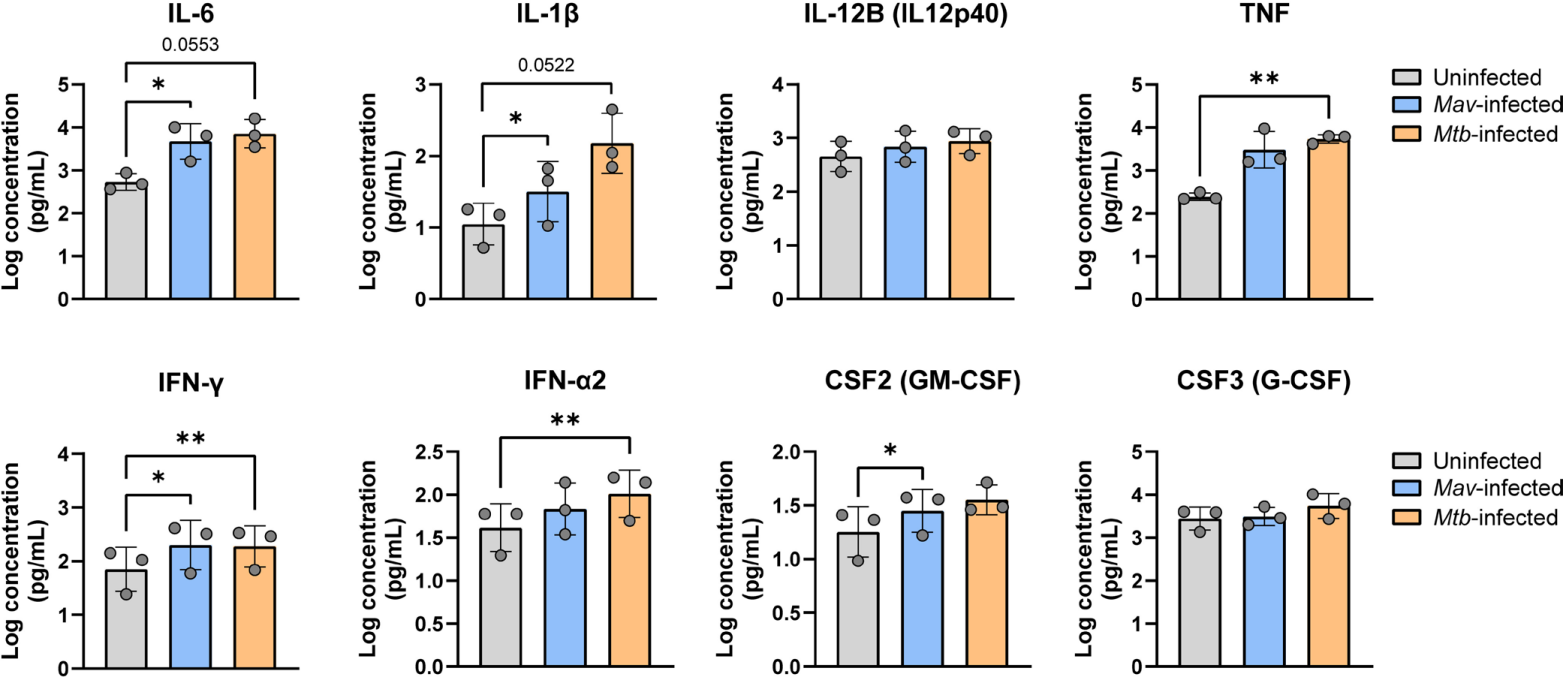
Cytokine production by *Mav*‐ and *Mtb*‐infected macrophages. Supernatants of *Mav*‐ and *Mtb* infected macrophages collected 24 h postinfection were assessed for IL‐6, IL‐1β, IL‐12B, TNF, IFN‐γ, IFN‐α2, CSF2 and CSF3 by the Luminex assay. Data represent the mean ± SD from different donors (*n* = 3). Dots represent the mean from duplicate wells of a single donor. Statistical significance was tested using a one‐way ANOVA with Tukey's multiple comparisons test. *p < 0.05 and **p < 0.005.

To validate the transcriptome analysis results of cytokine production (Figure S7A), secretion of a number of DEGs encoding cytokines in the supernatants of macrophages infected with *Mav* or *Mtb* 24 h postinfection was measured using the Luminex assay. Compared to uninfected controls, both *Mav* and *Mtb* infection resulted in the induction of IL‐6, IL‐1β, TNF, IFN‐γ, and to a lesser extent IL‐12B and IFN‐α2 (Figure 6). Induction of CSF2 and CSF3 by *Mav* or *Mtb* was not evident. Moreover, the transcriptome analysis between *Mav*‐ and *Mtb*‐infected macrophages indicated the higher expression of certain cytokines after *Mav* infection (Table 1, Figure S7). While *Mtb* rather than *Mav* tended to induce higher levels of certain cytokines, no statistically significant differences in cytokine production were observed between *Mav* and *Mtb* infections (Figure 6, Figure S7).

## 3 Discussion

There is a paucity of studies investigating the host‐pathogen interactions and host transcriptomic response in *Mav*‐infected primary human macrophages, cells crucial in immunity against *Mav* infection. Here, we report the first genome‐wide transcriptome analysis of macrophages infected with *Mav*, and directly cross‐reference these observations with *Mtb* infection. Our findings indicate that the transcriptional response to both infections largely overlaps, while some infection‐specific responses are at play. The shared response to *Mav* and *Mtb* primarily involved cytokine signaling responses and GPCR signaling. In contrast, when comparing *Mav* and *Mtb* to one another and uninfected controls, differences were observed in the regulation of lipid metabolism, IEG transcription factors, and the less‐explored GIMAPS. Overall, we found alterations in the host response to both mycobacteria, providing insights into the shared and distinctive host processes that may play a role in the intracellular control of *Mav* and *Mtb*, and which potentially offer targets for host‐directed therapy.

Macrophages have a leading role in mycobacterial killing, antigen presentation, and directing immune responses. Cytokines like TNF, IL‐1β, IL‐6, and IL‐10 produced by macrophages upon activation of pattern recognition receptors including Toll‐like receptors (TLR) are crucial in bridging the innate and adaptive immune responses to mycobacterial infection (Park et al. 2022). Consistent with previous findings, we observed a significant increase in pro‐inflammatory cytokines (*IL12B*, *IL23A*, *TNF*, *IL1B*, *IL6*, *CCL20*, *CSF3*, and *CSF2*) in macrophages within hours of *Mav* or *Mtb* infection (Agdestein et al. 2014; Greenwell‐Wild et al. 2002; Casey et al. 2015; Kawka et al. 2023). Some of these cytokines in turn regulate TLR transcription to create feedback loops (Wang et al. 2000). We found increased *TLR2* expression and decreased *TLR5* expression in macrophages up to 6 h postinfection with *Mav* and *Mtb*, as observed in prior studies (Agdestein et al. 2014; Wang et al. 2000; Ariel et al. 2019). In addition, *TLR3* and *TLR6* were downregulated in *Mav*‐ and *Mtb*‐infected macrophages. Our cytokine secretion data validate that cytokine responses are a common feature of both *Mav* and *Mtb* infections. At 2 h postinfection, however, differential expression analysis of infected macrophages showed a higher expression of pro‐inflammatory cytokines such as *TNF*, *CSF3*, and *IL6* in response to *Mav* as compared to *Mtb*, which did not result in differences in cytokine secretion patterns between *Mav*‐ and *Mtb*‐infected macrophages. Possibly, cytokine gene expression upon *Mtb* infection is slightly delayed as compared to *Mav* infection, which may be associated with the suggestion that mycobacterial virulence is inversely related to their ability to induce pro‐inflammatory cytokines as an immune evasion strategy (Strohmeier and Fenton 1999; Borrmann et al. 2011; Feng et al. 2020). Overall, these results reveal that early cytokine responses in macrophages are largely shared between *Mav* and *Mtb*, while minor pathogen‐specific variations may play a role in shaping the host immune environment.

Comparing the host transcriptomic response to *Mav* and *Mtb* revealed that both infections affected interferon signaling, which was more pronounced following *Mtb* infection. Both *Mav* and *Mtb* upregulated genes related to type II interferon (IFN) signaling. Interestingly, *Mav* affected type I IFN signaling only by upregulation of *IFNB1* (type I IFN), while *Mtb* induced the expression of genes downstream of type I IFN signaling (including *OAS2*, *MX1*, *MX2*, *ISG15*). In line with this, both *Mav* and *Mtb* seemed to induce secretion of IFN‐γ to a similar extent, whereas secretion of IFN‐α2 was slightly higher for *Mtb*‐infected macrophages. While type II IFN (i.e., IFN‐γ) is required for the resistance to mycobacteria, there is a lack of consensus on the role of type I IFNs in mycobacterial infections. In *Mav*‐infected mice, continuous IFN‐β infusion increased resistance, as evidenced by reduced bacterial loads (Denis 1991). In contrast, type I IFN worsens *Mtb* infections (Cooper et al. 2011), as shown by reduced bacterial loads in type I IFN receptor‐deficient mice, and increased bacterial burden and pathology associated with recruitment of permissive macrophages via CCL2 when IFN‐α/β was induced (Antonelli et al. 2010; Sabir et al. 2017). Remarkably, *CCL2* was more strongly downregulated in macrophages infected by *Mav* compared to *Mtb*. Moreover, type I IFN induces the immunosuppressive cytokine IL‐10 and suppresses IL‐1β production, resulting in the loss of protection against *Mtb* (Novikov et al. 2011; Mayer‐Barber et al. 2011; McNab et al. 2014). *IL1B* was more strongly upregulated in *Mav*‐infected cells compared to *Mtb* at 2 h. IL‐1β has a reciprocal control of type I IFN by controlling type I IFN‐induced accumulation of permissive macrophages at the site of infection through prostaglandin E2 (Mayer‐Barber et al. 2014). In line with the expression pattern of *IL1B*, *PTGS2*, which encodes for COX2 that mediates the production of prostaglandin E2, was more strongly upregulated by *Mav* compared to *Mtb* at 2 h. The disappearance of *IL1B* and *PTGS2* expression differences between *Mav* and *Mtb* at 6 h postinfection may explain the comparable cytokine secretion observed following both infections. Taken together, IFN signaling was affected by both *Mav* and *Mtb* infection, with considerable variation over time.

The host transcriptomic regulation by *Mav* and *Mtb* infection also involved many genes linked to lipid metabolism, with some clear differences between both infections. Fatty acids are the most energy‐dense substrates for energy production and are components of phospholipids in cell membranes (Remmerie and Scott 2018). When nutrients are in excess, fatty acids can be stored as triglycerides, together with cholesteryl esters, in lipid droplets, which can be accessed via lipophagy (hydrolysis of lipid droplets by lipo‐autophagosomes and lysosomes) or lipolysis (enzymatic hydrolysis of contents of cytosolic lipid droplets) during nutrient starvation (Zechner et al. 2017). We found that infection with *Mav* and *Mtb* commonly upregulated *HCAR2* (promotes lipid accumulation associated with *Mtb* survival) (Naz and Arish 2022), downregulated *FFAR4* (reduces lipid accumulation) (Stuttgen and Sahoo 2021), and upregulated GPR156 (increases lipid accumulation) (Kalam et al. 2023), indicating mycobacterial infection induces the accumulation and availability of lipids. Moreover, *Mav* and *Mtb* infections downregulated *GPR34* and closely related *GPR82* (both inhibit lipolysis) (Yasuda et al. 2023; Engel et al. 2011; Andreu et al. 2017; Liebscher et al. 2011) and upregulated *GPR84*, *GPR132*, and *GPR183* (all three involved in sensing fatty acids or cholesterol) (Wang et al. 2006; Hannedouche et al. 2011; Foster et al. 2019; Foo et al. 2023; Recio et al. 2018). In addition, the expression of *FFAR2* (i.e., *GPR43*), associated with inhibition of lipolysis (Al Mahri et al. 2022), varied in time and was more strongly downregulated by *Mav* compared to *Mtb* infection. Lipid metabolism is known to be crucial for *Mtb* survival during infections; *Mtb* stimulates intracellular lipid accumulation and access to cytosolic lipids by escaping the phagosome or promoting the transport of lipid droplets to mycobacteria‐containing vacuoles (van der Klugt et al. 2024), creating a nutrient‐rich environment that supports mycobacterial growth (Choi et al. 2024). While knowledge of the modulation of the host lipid metabolism during *Mav* infections is limited (Thirunavukkarasu et al. 2016), our findings suggest that lipid metabolism is also essential during *Mav* infections. Indeed, there is a clear association between lower body fat mass and the development of *Mav*‐LD (Kartalija et al. 2013; Tasaka et al. 2010), and increased fatty acid metabolism has been linked to disease progression (Choi et al. 2024), indicating that altered lipid metabolism is also involved during *Mav* infection. This is supported by *Mav*‐infected mice showing a correlation between increased fatty acid uptake and the formation of lipid‐rich foamy macrophages with the progression of pulmonary disease (Choi et al. 2024). Notably, *Mav* but not *Mtb* induced significant changes in the expression of phospholipases, which have a hydrolytic activity on host membrane phospholipids, resulting in the release of fatty acids for energy consumption, or anabolism of other lipids. These findings suggest that *Mav*, like *Mtb*, modulates lipid metabolism, possibly through different strategies in the battle between the host and mycobacteria for host lipids.

Other genes differentially regulated by *Mav* included several IEGs, which in turn regulate expression of genes associated with cell proliferation, differentiation, pro‐inflammatory cytokines and survival (Bahrami and Drablos 2016). Apoptosis of infected macrophages is a tightly regulated host defense mechanism that limits bacterial growth and promotes adaptive immunity, unlike necrosis, which involves cell lysis and bacterial release (Behar et al. 2011). The role of apoptosis in both *Mav* and *Mtb* infection is debated, as inhibition of apoptosis is recognized as a key strategy to impair host immunity (Keane et al. 2002; Liu et al. 2015; Bermudez et al. 2015; Early et al. 2011). However, mycobacteria can also benefit from the induction of apoptosis, which enables them to escape from dying cells to infect neighboring cells (Augenstreich et al. 2017; Aguilo et al. 2013; Fratazzi et al. 1997). Here, we observed that *Mav* infection induced expression of pro‐apoptotic neurotrophic factor receptor p75/*NGFR* 6 h postinfection (Bruno et al. 2022; Dechant and Barde 2002), although the NGF ligand was not detected. Increased expression of prosurvival *BCL2* and reduced expression of pro‐apoptotic *BAX* suggested that apoptosis was overall suppressed by both *Mav* and *Mtb*. This also indicates that IEGs did not specifically regulate apoptosis‐related gene expression in *Mav*‐infected macrophages. Instead, elevated expression levels of IEGs in *Mav*‐infected macrophages correlated positively with expression levels of pro‐inflammatory cytokines. Consistent with this, studies with *EGR1* knockout mice have previously demonstrated that transcription factor *EGR1* is involved in the production of IL‐1β and TNF (Silverman et al. 2001; Yan et al. 2000). Hence, *Mav* seems to elicit a stronger early transcriptional programme involving IEGs, which augments the proinflammatory cytokine response, as compared to *Mtb*.

Lastly, multiple GIMAPs were downregulated by both mycobacterial infections, and this downregulation was more pronounced during *Mav* infections. *GIMAP4* and *GIMAP7* were comparably silenced in macrophages by both *Mav* and *Mtb* 2 h postinfection. At 6 h postinfection, however, *Mav* showed a stronger suppression of *GIMAP1*, *GIMAP2*, *GIMAP5*, and *GIMAP6* expression compared to *Mtb*. To our knowledge, this is the first report that describes the differential expression of GIMAPs in human macrophages infected with mycobacteria. However, similar expression patterns were previously observed, although not described, in *Mav*‐infected macrophages (Agdestein et al. 2014). While the role of these proteins has mainly been described for the maintenance of lymphocytes (Pascall et al. 2018; Saunders et al. 2010; Schnell et al. 2006), GIMAPs are also thought to be important in intracellular trafficking, as well as autophagy and lysosome function (Limoges et al. 2021; Yao et al. 2022), processes considered important in immune defenses against mycobacteria. GIMAP2 is found on lipid droplets to which it recruits GIMAP7, suggesting a role for these GIMAPs in lipid droplet trafficking (Schwefel et al. 2010). Furthermore, mutations in GIMAP5, which resides on lysosomes, are linked to increased autoimmune susceptibility (Pascall et al. 2018), but its function in macrophages remains to be determined. GIMAP6 is involved in regulating efficient autophagy and facilitates antibacterial innate immunity by binding to and clearing pathogens (Pascall et al. 2018, 2013; Yao et al. 2022). Finally, GIMAP6 was downregulated in cattle infected with *Mav* subspecies paratuberculosis, while its role in disease susceptibility remains unknown (Thirunavukkarasu et al. 2014). Taken together, while it remains unclear what the exact roles of GIMAPs are during mycobacterial infection, the more profoundly reduced expression of these proteins observed upon *Mav* infection may indicate a stronger impairment of the macrophage's ability to manage the infection. More investigation into the role of GIMAPs during mycobacterial infection is desired and may reveal novel targets for HDT.

This study has several limitations that should be considered. Firstly, as a validation strategy, cytokine regulation was assessed by a Luminex, but other differences found in the transcriptomic data were not validated further by complementary analyses. Hence, the findings from this study require further validation. Secondly, the analysis focused exclusively on early time points postinfection, which represents only a snapshot of macrophage activity shortly after infection and may not reflect the longer‐term dynamic regulation of macrophage functions. Insufficient RNA yields at later time points (24 h postinfection) unfortunately limited our ability to assess gene expression over a prolonged time course. Finally, the bulk RNA‐seq data represent a heterogeneous population of infected and uninfected macrophages, and may therefore capture broader transcriptional changes occurring in the infection environment. Despite these limitations, a strength of this study was the use of RNA‐seq, which, unlike microarray studies performed previously on *Mav*‐infected cells (Agdestein et al. 2014; Blumenthal et al. 2005; Greenwell‐Wild et al. 2002; McGarvey et al. 2004), offers significant advantages including unbiased, genome‐wide transcriptome profiling of host gene expression without requiring preexisting genome sequence information. Additionally, our study directly compares *Mav* and *Mtb* infections across primary human macrophages from matched donors, providing relevant insights into the differential responses of macrophages to these two mycobacterial infections. This direct comparison between *Mav* and *Mtb* facilitates extrapolation of shared findings given the wealth of studies that have functionally validated RNA regulation by *Mtb*.

In conclusion, this study on the host transcriptomic regulation of the human macrophage response to Mav and *Mtb* infection reveals a significant overlap between these infections in gene expression patterns. However, also distinct effects were observed in macrophage gene expression, being particularly pronounced during *Mav* infection. The functional implications of these expression patterns remain to be determined, in which our results provide direction to further explore host–pathogen interactions during *Mav* and *Mtb* infections.

## 4 Experimental Procedures

### 4.1 Cell Culture

Buffy coats were collected from healthy anonymous Dutch adult donors after written informed consent (Sanquin Blood Bank, Amsterdam, The Netherlands). Primary human macrophages were obtained as previously described (Kilinc et al. 2022). In short, CD14+ monocytes were isolated from peripheral blood mononuclear cells using density gradient centrifugation with Ficoll (Pharmacy, LUMC, the Netherlands) and subsequently magnetic‐activated cell sorting (MACS) with anti‐CD14‐coated microbeads (Miltenyi Biotec, Auburn, CA, USA). Purified CD14+ monocytes were cultured for 6 days at 37°C/5% CO_2_ in Gibco Dutch modified Roswell Park Memorial Institute (RPMI) 1640 medium (ThermoFisher Scientific, Landsmeer, the Netherlands) supplemented with 10% fetal calf serum (FCS), 2 mM L‐glutamine (PAA, Linz, Austria), 100 units/mL penicillin, 100 μg/mL streptomycin, and 50 ng/mL macrophage colony‐stimulating factor (M‐CSF, R&D Systems, Abingdon, UK) for anti‐inflammatory M2 macrophage differentiation. Cytokines were refreshed at day 3 of differentiation. One day prior to experiments, macrophages were harvested and seeded into flat‐bottom 96‐well plates (30,000 cells/well), if not indicated otherwise, in complete RPMI medium without antibiotics or cytokines. Macrophage differentiation was validated based on cell surface marker expression (anti‐human CD163‐PE, CD14‐PE‐Cy7, and CD1a‐Alexa Fluor 647 (1:20) from Biolegend (Amsterdam, the Netherlands) and anti‐human CD11b‐BB515 (1:20) from BD Biosciences) using flow cytometry and secretion of cytokines (IL‐10 and IL‐12) following 24 h stimulation of cells with 100 ng/mL lipopolysaccharide (InvivoGen, San Diego, United States) using ELISA.

### 4.2 Bacterial Cultures

*Mav*‐Wasabi (laboratory strain 101) and *Mtb*‐Venus (H37Rv) were cultured in Difco Middlebrook 7H9 broth supplemented with 0.2% glycerol, 10% Middlebrook ADC (albumin, dextrose, catalase), and 0.05% Tween‐80 (Kilinc et al. 2022; Korbee et al. 2018). Prior to experiments, bacterial concentrations were determined by measuring the optical density at 600 nm (OD_600_).

### 4.3 Bacterial Infection of Cells

Infection experiments were performed as previously described (Kilinc et al. 2022; Korbee et al. 2018). Briefly, 1 day before infection, *Mav* and *Mtb* cultures were diluted to a density corresponding with early log‐phase growth, OD_600_ of 0.25. On the day of macrophage infection, bacterial suspensions were diluted in antibiotic‐free cell culture medium to consistently infect cells with a multiplicity of infection (MOI) of 10. The accuracy of the MOI was verified using a standard CFU assay. Following inoculation of the cells, plates were centrifuged for 3 min at 130 RCF and incubated for 1 h at 37°C/5% CO_2_. Cells were then treated with cell culture medium supplemented with 30 μg/mL gentamicin for 10 min to inactivate and remove residual extracellular bacteria, after which the medium was refreshed with medium containing 5 μg/mL gentamicin sulfate before cells were incubated at 37°C/5% CO_2_ until indicated timepoints. Following incubation, supernatants were either stored at −20°C for Luminex assay or discarded. To assess the percentage of infected cells, cells were washed, fixed with 1% paraformaldehyde, and measured on a BD Accuri C6 Plus flow cytometer before analysis using FlowJo v10 software (BD Biosciences). Alternatively, cells were lysed using 100 μL of lysis buffer (H2O + 0.05% SDS) for the determination of intracellular bacterial burden using a CFU assay or lysed for RNA extraction as described below.

### 4.4 RNA Isolation and Sequencing

Total RNA was extracted from *Mav*‐ or *Mtb* infected macrophages seeded in a flat bottom 6‐wells plate (900,000 cells/well) with 350 μL TRIzol reagent (Thermo Fisher Scientific) and using the Direct‐zol RNA miniPrep kit (Zymo Research, Leiden, Netherlands) according to the manufacturer's protocol. Samples were diluted in 25 μL RNA‐free water, and the total RNA concentration of each sample was quantified using DeNovix DS‐11 Spectrophotometer (ThermoFisher Scientific). Nanodrop (ThermoFisher Scientific) was used to determine RNA purity. Gene expressions were profiled using the NovaSeq 6000 platform (Illumina, San Diego, CA, USA) by GenomeScan (Leiden, Netherlands).

### 4.5 Data Processing and Analysis

RNA‐Seq files were processed using the opensource BIOWDL RNAseq pipeline v5.0.0 (https://zenodo.org/record/5109461#.Ya2yLFPMJhE) developed at the LUMC. This pipeline performs FASTQ preprocessing (including quality control, quality trimming, and adapter clipping), RNA‐Seq alignment, read quantification, and optionally transcript assembly. FastQC was used for checking raw read QC. Adapter clipping was performed using Cutadapt (v2.10) with default settings and standard lllumina universal adapter “AGATCGGAAGAG”. RNA‐Seq reads' alignment was performed using STAR (v2.7.5a) on GRCh38 human reference genome. umi_tools (v1.1.1) was used to remove PCR duplicates detected with UMIs. The gene read quantification was performed using HTSeq‐count (v0.12.4) with setting “–stranded = reverse”. The gene annotation used for quantification was Ensembl version 111. Using the gene read count matrix, CPM was calculated per sample on all annotated genes. Genes with a higher log2CPM than 1 in at least 25% of all samples are kept for downstream analysis.

For the differential gene expression analysis and PCA plot creation, dgeAnalysis R‐shiny application (https://github.com/LUMC/dgeAnalysis/tree/v1.4.4) was used. EdgeR (v3.34.1) with TMM normalization was used to perform differential gene expression analysis using donor as *covariate*. Genes with log2(fold change) ≥ 1.5 or ≤ −1.5 and Benjamini and Hochberg false discovery rate (FDR) adjusted *p*‐values < 0.05 were designated as differentially expressed genes (DEGs).

### 4.6 Functional Enrichment Analysis

To classify the functions of the DEGs, functional enrichment analysis and clustering of biological pathways was performed through the use of QIAGEN Ingenuity Pathway Analysis (IPA) (QIAGEN Inc., https://digitalinsights.qiagen.com/IPA) (Kramer et al. 2014). In addition, enrichment of Gene Ontology (GO) categories biological process, cellular component and molecular function was analyzed. Enrichment with an adjusted P value of < 0.05 was considered significant. The protein–protein interaction (PPI) networks of DEGs were predicted using the Search Tool for the Retrieval of Interacting Genes (STRING) database.

### 4.7 Cytokine Secretion

Collected supernatants of uninfected or *Mav*‐ and *Mtb*‐infected macrophages were filtered in FiltrEX 96‐wells filter plates (Corning Costar) with pore size 0.2 μm to remove bacteria. The concentration of IL‐6, IL‐1β, TNF, IFN‐γ, IL‐12B, IFN‐α2, CSF2, and CSF3 was measured by diluting the supernatants four times with Luminex Assay buffer (Bio‐Rad, Hercules, CA, USA). Next, the Bio‐Plex Pro Human Cytokine 48‐plex Assay (Bio‐Rad) was performed according to the manufacturer's instructions. Samples were measured on a Bio‐Plex 200 System (Bio‐Rad). Per analyte, a lower and upper limit of detection was determined with standard curves. Concentrations measured below the assays' detection limit were set to 1 pg/mL, and those measured over the detection limit were set to the maximum quantifiable pg/mL per analyte.

## Author Contributions

**Gül Kilinç:** conceptualization, methodology, investigation, formal analysis, visualization, writing – original draft, writing – review and editing. **Robin H. G. A. van den Biggelaar:** supervision, writing – review and editing. **Tom H. M. Ottenhoff:** funding acquisition, writing – review and editing. **Leon H. Mei:** methodology, investigation, data curation. **Anno Saris:** conceptualization, supervision, writing – review and editing

## Disclosure

Copyright Statement: The graphical abstract was created using BioRender.com. Pathway and network analyses shown in Figure 3B,C were generated using Ingenuity Pathway Analysis (IPA, QIAGEN Inc.). Protein–protein interaction analysis shown in Figure 5C was generated using the STRING database.

## Ethics Statement

Human blood samples were isolated from buffy coats obtained from healthy donors after written informed consent (Sanquin, Amsterdam, the Netherlands). The biological samples were sourced ethically, and their research use was in accordance with the terms of the informed consents under an IRB/EC approved protocol. The use of blood samples was approved by the Sanquin Ethical Advisory Board and in accordance with the Declaration of Helsinki.

## Conflicts of Interest

The authors declare no conflicts of interest.

## Supporting information


**Data S1:** mmi70045‐sup‐0001‐Supinfo.pdf.

**Table S1:** Gene expression value of *Mav‐* and *Mtb*‐infected macrophages compared to uninfected controls.

## Data Availability

The data that support the findings of this study are openly available in ArrayExpress at https://www.ebi.ac.uk/biostudies/arrayexpress, reference number E‐MTAB‐15473.
