# Supplementary material for: Comparative Transcriptomic Analysis of Human Macrophages During Mycobacterium avium Versus Mycobacterium tuberculosis Infection
Source: Mol Microbiol. 2026 Jan 5;125(3):185–202. doi: 10.1111/mmi.70045 (PMC12956043; doi:10.1111/mmi.70045)
Supplement: Supplementary file 1 — Data S1: mmi70045‐sup‐0001‐Supinfo.pdf. [file MMI-125-185-s001.pdf]

Comparative transcriptomic analysis of human macrophages during *Mycobacterium avium* versus *Mycobacterium tuberculosis* infection

Gül Kilinç[1], Robin H. G. A. van den Biggelaar[1*], Tom H. M. Ottenhoff[1], Leon H. Mei[2] and Anno Saris[1]

[1] Leiden University Center for Infectious Diseases, Leiden University Medical Center, Leiden, The Netherlands
[2] Department of Biomedical Data Sciences, Leiden University Medical Center, Leiden, The Netherlands.
*Corresponding author

E-mail addresses:

Gül Kilinç:	g.kilinc@lumc.nl
Robin H. G. A. van den Biggelaar:	r.h.g.a.van_den_biggelaar@lumc.nl
Tom H. M. Ottenhoff:	t.h.m.ottenhoff@lumc.nl
Leon H. Mei:	h.mei@lumc.nl
Anno Saris:	a.saris@lumc.nl

Supplementary information

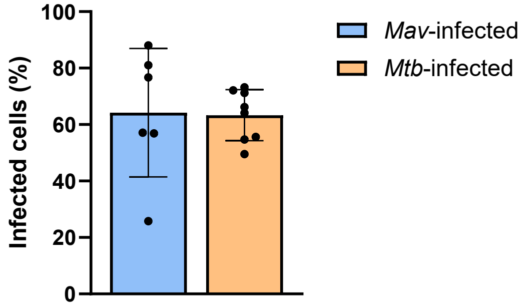

Supplementary Figure 1. Representative infection rates of macrophages infected with *Mav* or *Mtb*. M2 macrophages were infected for 1 hour with either fluorescently-labeled *Mav*-Wasabi or *Mtb*-Venus. After infection, cells were washed and fixed to determine the percentages of infected cells by flow cytometry. Each dot represents a different donor tested in triplicate (n = 6 for *Mav* and n = 8 for *Mtb*). Bars and error bars show the mean ± standard deviation (SD). The donors used to generate the data were different from those used in the RNA-seq analysis. *Mav* data were previously published (adapted from Kilinç et al., Frontiers in Cellular and Infection Microbiology, 2022, under CC-BY license).

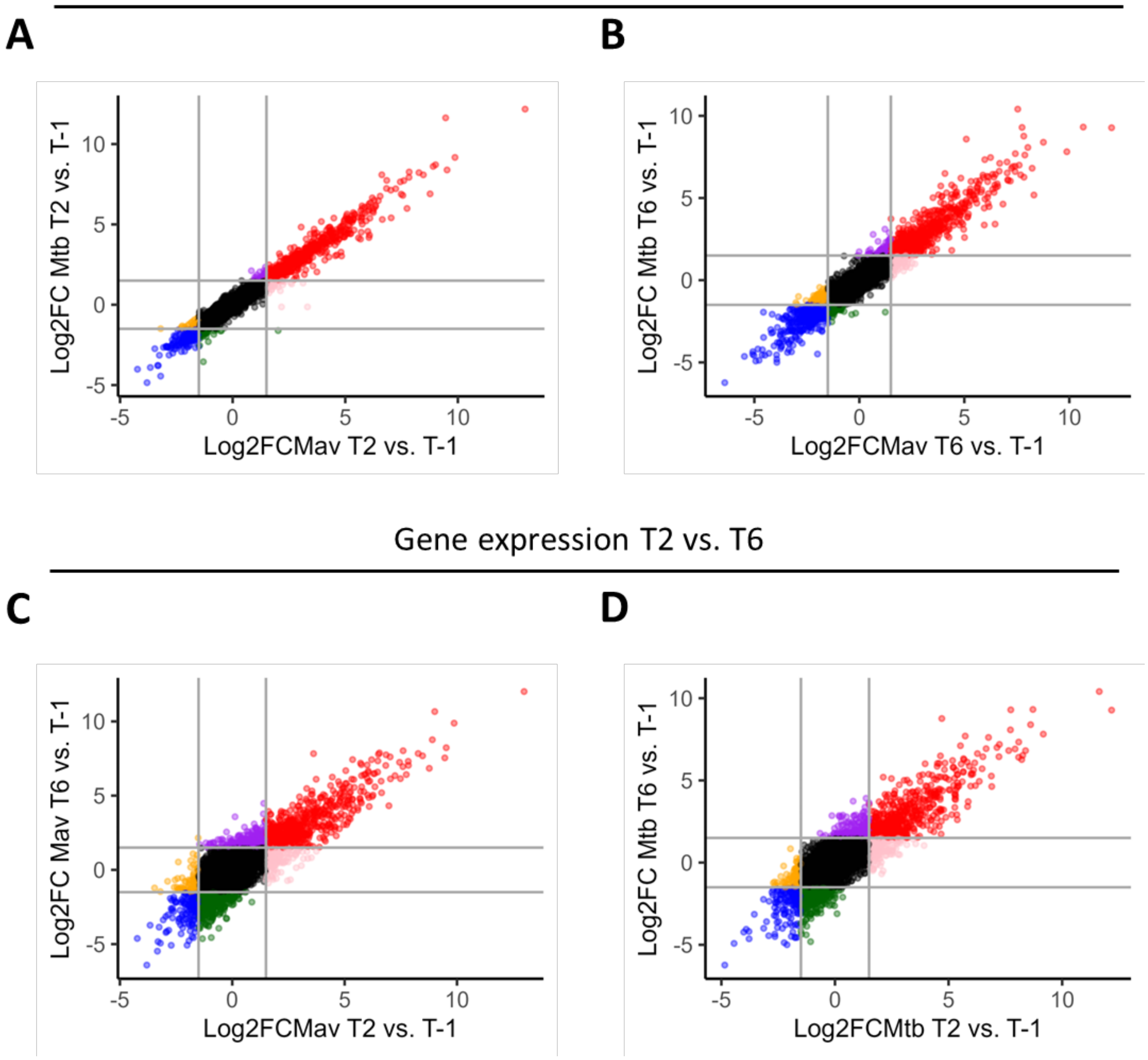

Supplementary Figure 2. Transcriptomic response of primary human macrophages infected with *Mav* or *Mtb* at 2 or 6 hours post-infection and uninfected controls.
(A-B) Scatterplot showing gene expression levels (Log2FC ≥ 1.5 or ≤ 1.5) of macrophages infected with *Mav* vs. *Mtb* at 2 hours (A) or 6 hours (B) post-infection compared to uninfected controls. Genes with Log2FC ≥ 1.5 and Log2FC ≤ 1.5 by both *Mav* and *Mtb* are expressed red and blue, respectively. (C-D) Scatterplot showing gene expression levels (Log2FC ≥ 1.5 or ≤ 1.5) of macrophages 2 hours vs. 6 hours post-infected with *Mav* (C) or *Mtb* (D) compared to uninfected controls. Genes with Log2FC ≥ 1.5 and Log2FC ≤ 1.5 by both timepoints post-infection are expressed red and blue, respectively.

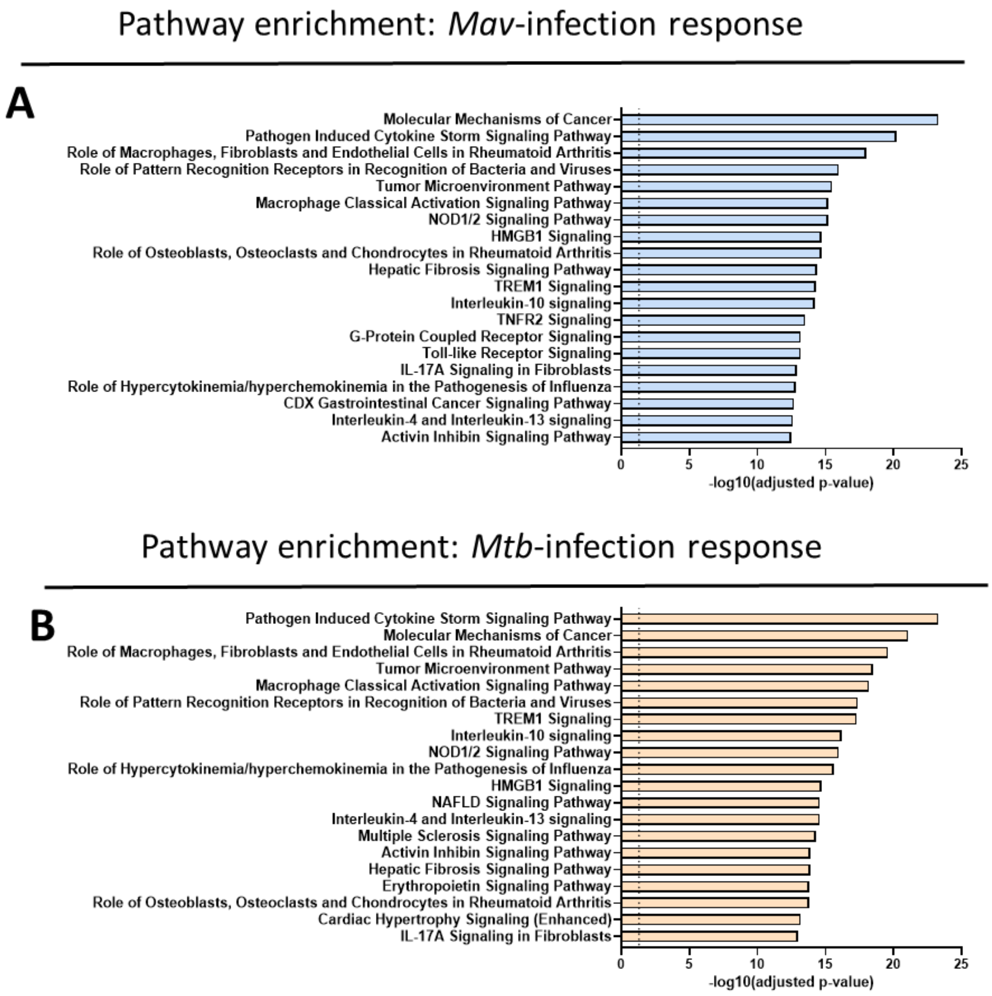

Supplementary Figure 3. Pathway enrichment analysis of the whole transcriptomic response induced by either *Mav* or *Mtb*.

(A-B) The top 20 most significantly enriched IPA pathways based on the whole host transcriptomic response consisting of all genes down- or upregulated in macrophages infected with *Mav* (A) or *Mtb* (B) compared with uninfected controls. The enriched pathways were ranked by -log 10 p-value of gene enrichment.

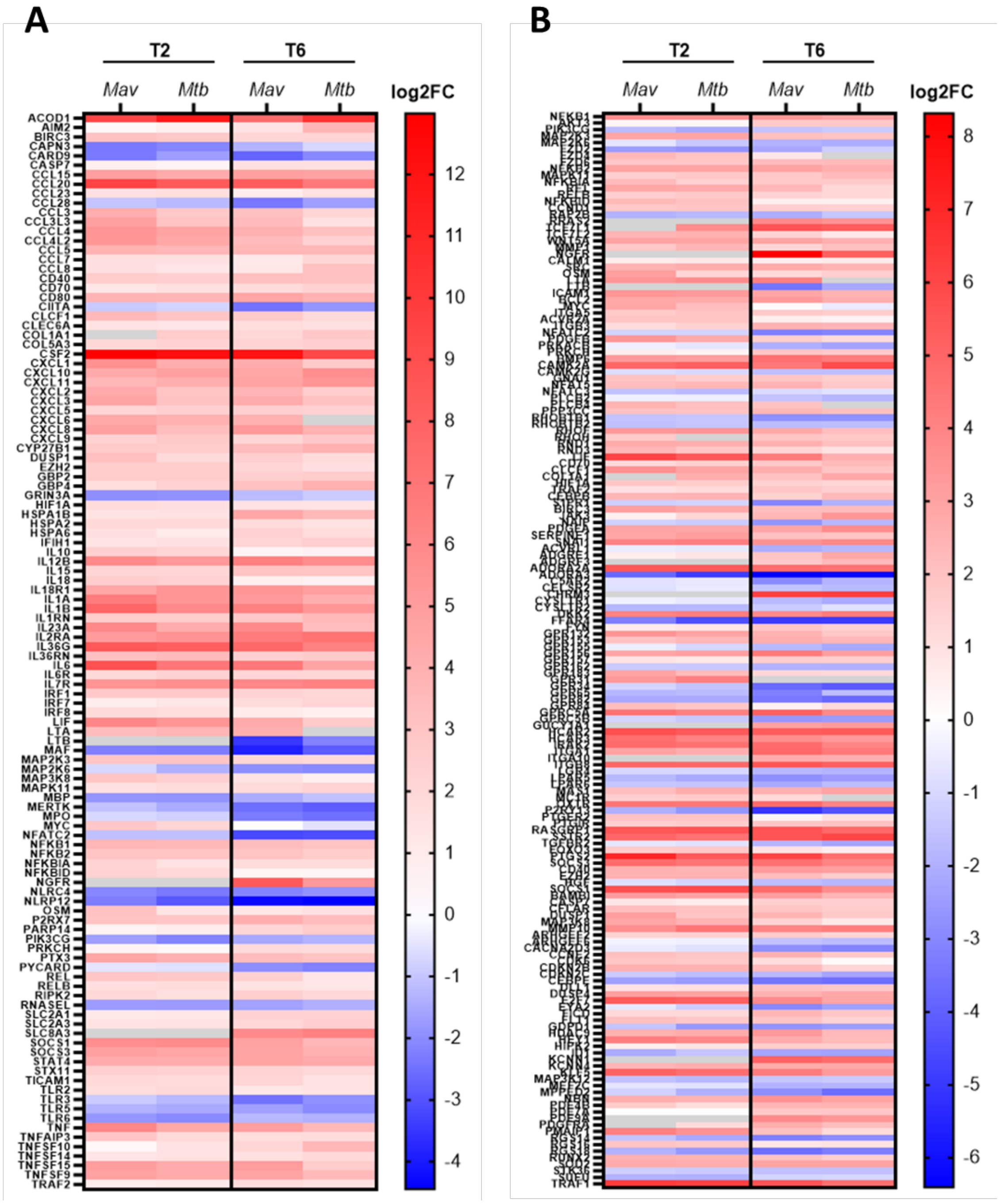

Supplementary Figure 4. Expression patterns of DEGs belonging to the cytokine response or disease-related response commonly induced by *Mav* or *Mtb.*

(A-B) Heatmap showing the expression patterns of 114 DEGs belonging to the cytokine response (A) or 164 DEGs associated with disease pathways (B) commonly induced by *Mav* and *Mtb* in comparison to uninfected controls. Grey box indicates no expression values could be determined.

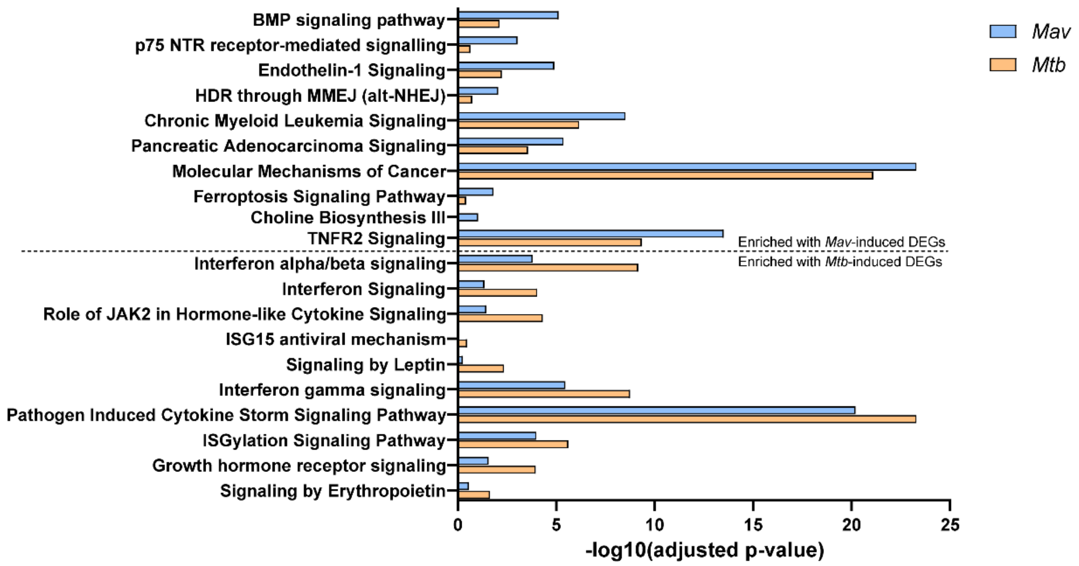

Supplementary Figure 5. Pathway analysis reveals only subtle differences in host signaling between *Mav*- and *Mtb*-infected macrophages.

The top 10 most significantly enriched IPA pathways based on the genes exclusively affected in macrophages infected with either *Mav* (561 DEGs, above dotted line) or *Mtb* (323 DEGs, below dotted line) compared with uninfected controls, also showing the -log 10 p-value of gene enrichment values for the other infection. The enriched pathways were ranked by -log 10 p-value of gene enrichment.

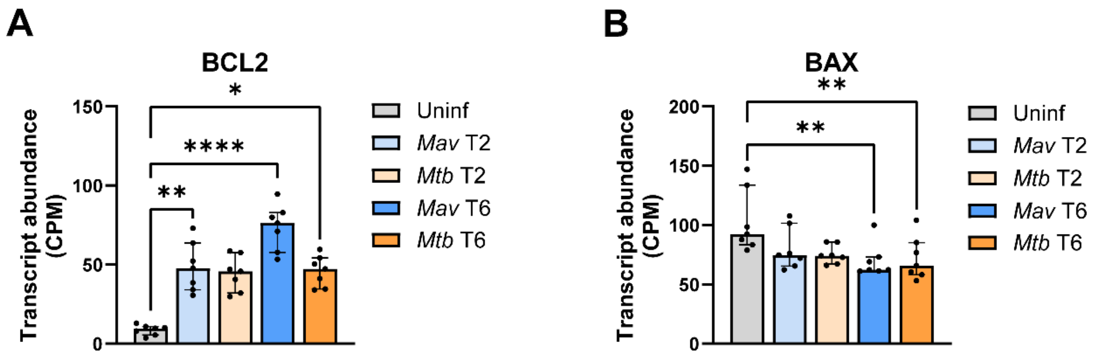

Supplementary Figure 6. Expression profiles of *BCL2* and *BAX* show tendency towards host survival in infected macrophages.

(A-B) Transcript levels (count per million; CPM) of *BLC2* (A) and *BAX* (B) in uninfected (grey), and *Mav* (blue shaded)- and *Mtb* (orange shaded)-infected macrophages at 2 and 6 hours post-infection. Differences were statistically significant by a Friedman test with Dunn's multiple comparison test. *p < 0.05, **p < 0.005 and ****p < 0.0001.

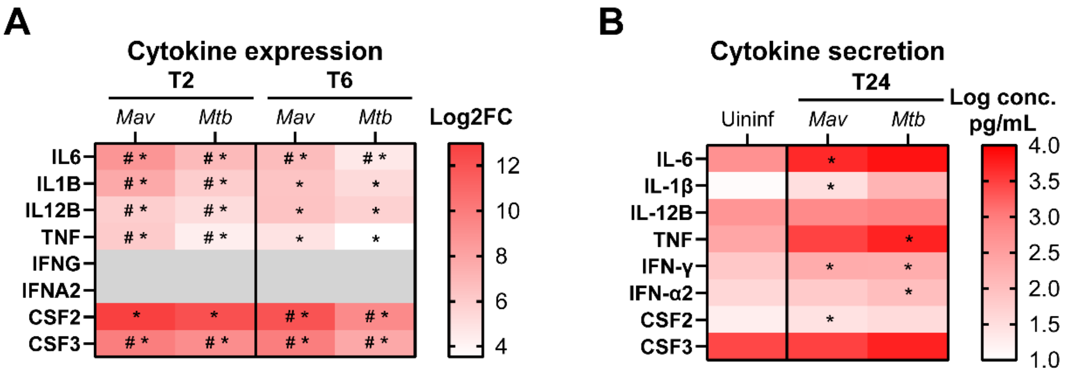

Supplementary Figure 7. Validation of cytokine expression by assessment of cytokine secretion by macrophages infected with *Mav* or *Mtb*.

(A) Heatmap showing the expression patterns of *IL6*, *IL-B*, *IL12B*, *TNF*, *IFNG*, *IFNA2*, *CSF2* and *CSF3* that were differentially regulated in *Mav*-infected macrophages at 2 (T2) and/or 6 (T6) hours post-infection, compared to uninfected or *Mtb*-infected macrophages. (B) Heatmap showing secretion of IL-6, IL-1β, IL-12B, TNF, IFN-γ, IFN-α2, CSF2 and CSF3 measured in supernatants of *Mav*- and *Mtb* infected macrophages collected 24 hours post-infection by the Luminex assay. Shown is the mean from three donors. Asterisk (*) indicates differential expression/secretion when compared to uninfected controls, whereas number sign (#) indicates differential expression/secretion between *Mav* and *Mtb*.

Supplementary Table 1. Gene expression value of *Mav*- and *Mtb*-infected macrophages compared to uninfected controls

Excel file with complete gene expression data.

Supplementary Table 2. Genes differentially expressed between *Mav* and *Mtb* not associated with a STRING-node.

**2 hours post-infection**			**DEG vs. uninfected**	
**Gene**	**Log2FC (*Mav* vs. *Mtb*)**	**p-value (adj)**	***Mav***	***Mtb***
OSR2	1,34	9,89E-03	Up	Up
**6 hours post-infection**			**DEG vs. uninfected**	
**Gene**	**Log2FC (*Mav* vs. *Mtb*)**	**p-value (adj)**	***Mav***	***Mtb***
GPR65	-1,98	2,93E-03	Down	Down
ENSG00000289424	-1,84	2,93E-03	Down	-
SNAI3	-1,84	2,93E-03	-	-
RAB42	-1,75	2,93E-03	Down	Down
FFAR2	-1,61	2,43E-02	-	Up
LRG1	-1,53	4,61E-02	-	Up
SDHAF1	-1,53	1,06E-02	Down	-
DUSP15	2,97	1,95E-02	Up	Up
CDC25A	1,72	1,06E-02	Up	-
PALLD	1,64	2,58E-03	Up	-
DUSP8	1,59	3,75E-02	Up	Up
ZNF433	1,58	9,12E-03	Up	-
ANKRD1	1,56	6,19E-03	Up	Up
C5orf34	1,54	3,58E-02	Up	-
